# Supplementary material for: Universal health coverage—Exploring the what, how, and why using realist review
Source: PLOS Glob Public Health. 2025 Mar 18;5(3):e0003330. doi: 10.1371/journal.pgph.0003330 (PMC11918392; doi:10.1371/journal.pgph.0003330)
Supplement: S1 File — (DOCX) [file pgph.0003330.s001.docx]

S1 File: Search Strategies

| Databases | Search strategy | Access date | Total number of articles |
| --- | --- | --- | --- |
| PubMed | ("Universal Health Coverage"[Title/Abstract] OR "Universal Coverage"[Title/Abstract] OR "Universal Health Care"[Title/Abstract] OR "Universal HealthCare"[Title/Abstract]) | 4/01/2024 | 8,426 |
| Scopus | (TITLE-ABS-KEY("Universal Health Coverage") OR TITLE-ABS-KEY("Universal Coverage") OR TITLE-ABS-KEY("Universal Health Care") OR TITLE-ABS-KEY("Universal HealthCare")) | 4/01/2024 | 12,148 |
| Web of Science | ((((TI=("Universal Health Coverage" )) OR TI=( "Universal Coverage" )) OR TI=("Universal Health Care" )) OR TI=("Universal HealthCare")) | 4/01/2024 | 2,658 |
| EMBASE | 'universal health care':ti OR 'universal coverage':ti OR 'universal health coverage':ti OR 'universal healthcare':ti | 4/01/2024 | 2,542 |
