# Supplementary material for: Universal health coverage—Exploring the what, how, and why using realist review
Source: PLOS Glob Public Health. 2025 Mar 18;5(3):e0003330. doi: 10.1371/journal.pgph.0003330 (PMC11918392; doi:10.1371/journal.pgph.0003330)
Supplement: S2 File — (DOCX) [file pgph.0003330.s002.docx]

S2 File: Characteristics of articles

| **Author** | **Article category and study design** | **Country** | **Used for** |
| --- | --- | --- | --- |
| Davis K/2001 | UHC: lessons from experience: Methods not specified | USA | What is UHC? |
| Pan American Health Organization & World Health Organization | Universal Health Day 2022: Report | Non-specific | What is UHC? |
| Haakenstad A et al/2022 | Performance of the Healthcare Access and Quality Index: Quantitative study | 204 countries and territories | What is UHC? |
| Luankongsomchit V et al/2023 | How Many People Experience Unsafe Medical Care: Quantitative study | Thailand | What is UHC? |
| Hui C/2023 | Undetectable= Untransmittable= Universal Access: Narrative review | Australia, USA, Canada, Vietnam, UK, Nepal, New Zealand, Zambia, South Africa, Thailand, Kenya, Germany | What is UHC? |
| Traynor K/2004 | Calls for Universal Health Care Coverage: News | USA | What is UHC? |
| Eastman P/2004 | Calls for Universal Health Insurance: News | USA | What is UHC? |
| McIntyre D et al/2008 | Beyond fragmentation and towards UHC: Qualitative analysis | Ghana, South Africa, Tanzania | What is UHC? |
| Sukmanee J et al/2023 | Impact of UHC on OOP: Quantitative study | Thailand | What is UHC? |
| Naidoo S et al/2023 | Manager’s knowledge and attitudes toward UHC: Qualitative study | South Africa | What is UHC? |
| Muinde JVS and Prince RJ/2023 | UHC and Debate about rights, solidarity and inequality: Qualitative study | Kenya | What is UHC? |
| Wagstaff A et al/2016 | Measuring progress towards UHC: Quantitative study | 24 developing countries | What is UHC? |
| Beattie A et al/2016 | Progress towards UHC lessons learned: Methods not specified | Asia and Pacific | What is UHC? |
| Savedoff WD et al/2012 | Political and economic aspects of the transition to UHC: Methods not specified | Nonspecific | What is UHC? & Why UHC? |
| Sanadgol A et al/2022 | Role of non-governmental organizations on UHC: Qualitative study | Iran | What is UHC? |
| Ng M et al/2014 | A metric for monitoring UHC: review | Not specific | What is UHC? |
| Stevens A et al/2023 | Quality & equity as shared agenda of UHC: commentary | Not specific | What is UHC? |
| Glassman A et al/2016 | Health benefit packages: No specified method | Not specific | What is UHC? |
| World Health Organization/2021 | Principles of health benefit packages: WHO document | Not specific | What is UHC? |
| Mangoya D et al/2023 | Health benefit packages selection for UHC: review | Low- and middle-income countries | What is UHC? |
| Eregata GT et al/2020 | Revision of Health benefit packages: Review | Ethiopia | What is UHC? |
| Ochalek J et al/2018 | Health benefit packages: No specified method | Malawi | What is UHC? |
| Hogan DR et al/2018 | Monitoring UHC: Quantitative study | 183 countries | What is UHC? |
| Lozano R et al/209 | Monitoring UHC: Quantitative study | 204 countries | What is UHC? |
| Mchenga M et al/2022 | Developing Malawi's UHC index: Quantitative study | Malawi | What is UHC? |
| Liu X et al/2021 | Progress towards UHC: Qualitative study | China | What is UHC? |
| Li Y et al/2023 | Projection towards UHC: Quantitative study | China | What is UHC? |
| Nambiar D et al/2020 | Monitoring UHC reforms: Delphi method | India | What is UHC? |
| Reddock J/2017 | Parameters to evaluate UHC: No specified method | Not specific | What is UHC? |
| Goodman L et al/2023 | Eye health service as UHC element: Editorial | High income countries | What is UHC? |
| Winkelmann J et al/2023 | UHC cannot be universal without oral health: Editorial | Not specific | What is UHC? |
| Mathur M et al/2015 | UHC for oral health: Editorial | Not specific | What is UHC? |
| World Health Organization and World Bank/2023 | Tracking UHC: Report | worldwide | What is UHC? |
| Kalita et al/2023 | Barriers and strategies of UHC: Qualitative study | India | What is UHC? |
| Arhin K et al/2023 | Efficiency of health system in achieving UHC: Quantitative study | Sub-Saharan Africa | What is UHC? |
| Tao W et al/2020 | Achievements and challenges towards UHC: Mixed method study | China | What is UHC? |
| Kaiser AH et al/2023 | Progress towards UHC: Quantitative study | Cambodia | What is UHC? |
| Okoroh et al/2020 | Health insurance: Quantitative study | Ghana | What is UHC? |
| Stewart BT et al/2021 | Health insurance: Quantitative study | Ghana | What is UHC? |
| Haas et al/2012 | Indicators of UHC: Mixed-method study | Bangladesh, Ethiopia, Peru, Uganda, and Vietnam | What is UHC? |
| Saksena P et al/2014 | Financial risk protection and UHC: Review | Not specific | What is UHC? |
| World Health Organization/2005 | Sustainable health financing, universal coverage and social health insurance: Resolutions and decisions | URGES Member States | How UHC works? |
| Koohpayehzadeh J et al/2021 | Best practices in achieving UHC: a scoping review | Iran | What is UHC? |
| Chapman AR/2018 | Monitoring UHC: Commentary | Not specific | What is UHC? |
| The Lancet Public Health/2019 | UHC: Editorial | Not specific | What is UHC? |
| O'Connell T et al/2014 | UHC: viewpoint | Not specific | Why UHC? |
| UNICEF/2012 | Advancing equitable social health protection to achieve UHC: Document | Asia and Africa | Why UHC? |
| Abiiro GA & De Allegri M/2015 | UHC from multiple perspectives: Debate | Not specific | What is UHC? |
| Shaikh BT & Ali N/2023 | UHC: Debate | Pakistan | How UHC works? |
| Lagomarsino G et al/2012 | Moving towards UHC: Review | 9 developing countries in Asia and Africa | How UHC works? |
| Iqbal MH/2019 | Does UHC reduce disparities: Quantitative study | Bangladesh | How UHC works? |
| Donnelly PD et al/2019 | Single-payer, multiple-payer, and state-based financing of health care: Editorial | USA | How UHC works? |
| Fox A & Poirier R/2018 | Different models of UHC: comparative study | 17 High income countries | How UHC works? |
| McKee M/2019 | Single-payer health care system: Comment & response | Canada | How UHC works? |
| Ivers N et al/2018 | Single-payer health insurance: Editorial comment | Canada | How UHC works? |
| Kuo L-w et al/2019 | Single-payer UHC: Quantitative study | Taiwan | How UHC works? |
| Cahin C & Dossou J-P/2021 | The way to UHC: Commentary | Sub-Saharan Africa | How UHC works? |
| Ipinnimo T et al/2023 | Comparing old and recent national health insurance: Review | Nigeria | How UHC works? |
| Luo Y et al/2023 | Towards UHC: Quantitative study | China | How UHC works? |
| Angeles MR et al/2000 | Medicare and UHC: Qualitative study | Australia | How UHC works? |
| Dobrosak C & Dugdale P/2021 | Reregulation of private hospital insurance: Qualitative study | Australia | How UHC works? |
| Gilory J et al/2023 | Disability workforce and planning process: scoping review | Australia | How UHC works? |
| Merga BT et al/2022 | Health insurance coverage: Quantitative study | Ethiopia | How UHC works? |
| Fang H et al/2019 | Social health insurance: review of records | China | How UHC works? |
| Yokobori Y et al/2023 | Social protection: review | Low- and middle-income countries | How UHC works? |
| Hasan SS et al/2022 | Sehat Sehulat Program: commentary | Pakistan | How UHC works? |
| Ayub A et al/2018 | Progress toward UHC: special communication | Pakistan | How UHC works? |
| Short SD et al/2021 | Reciprocal health care agreement: case study | Australia & South Korea | How UHC works? |
| Nguyen TM et al/2023 | Value-based health care: viewpoint | Australia | How UHC works? |
| Dohmen P et al/2023 | Value-based health care: implementation study | Kenya | How UHC works? |
| Wilson DR et al/2023 | Digital financing services: Qualitative study | Kenya & Rwanda | How UHC works? |
| van de Vijver S et al/2023 | Digital health for all: policy and practice | Not specific | How UHC works? |
| Bold B et al/2023 | Role of artificial intelligence: Editorial | Mongolia | How UHC works? |
| Martinez-Millana A et al/2022 | Artificial intelligence: overview of systematic review | Not specific | How UHC works? |
| Arhin K et al/2022 | Effect of PHC expenditure on UHC: Quantitative study | Sub-Saharan Africa | How UHC works? |
| Van Weel C & Kidd MR/2018 | Strengthening PHC to achieve UHC: concept analysis | Not specific | How UHC works? |
| Sacks E et al/2020 | Communities, UHC & PHC: policy and practice | worldwide | How UHC works? |
| Assefa Y et al/2020 | PHC contribution to UHC: Mixed-method analysis | Ethiopia | How UHC works? |
| Hossain AD et al/2023 | The role of civil society: commentary | Uganda | How UHC works? |
| Jayaraman A & Fernandez A/2023 | The role of civil society: perspective | India | How UHC works? |
| Levine AC et al/2023 | The role of civil society: Qualitative study | Developing countries | How UHC works? |
| Van Niekerk et al/2023 | Social innovation in health: Qualitative study | Philippines, Malawi, and Colombia | How UHC works? |
| Shilton T & Barry MM/2022 | The role of health promotion: commentary | Not specific | How UHC works? |
| Olu O et al/2019 | Community participation and private sector: meeting report | Africa | How UHC works? |
| Knaul FM et al/2012 | The quest for universal health coverage: achieving social protection for all in Mexico | Mexico | How UHC works? |
| Saengtabtim K et al/2023 | The impact of UHC: Quantitative study | South-east Asia & Western Pacific Region | Why UHC? |
| Galvani AP et al/2022 | Lives and costs saved during COVID-19: Quantitative study | USA | Why UHC? |
| Hajjar K et al/2023 | Association between UHC and diseases burden: Quantitative study | 132 countries | Why UHC? |
| Imran et al/2022 | Curbing antimicrobial resistance: Editorial | Pakistan | Why UHC? |
| Moreno-Serra R & Smith PC/2022 | Impact of UHC on population health: review | Not specific | Why UHC? |
| Yuki M/2016 | UHC and health outcomes: report | OECD countries | Why UHC? |
| Ranabhat CL et al/2018 | The impact of UHC on life expectancy: Quantitative study | 193 united nation member countries | Why UHC? |
| Kruk ME et al/2018 | Mortality due to low quality health system: Quantitative study | 137 countries | Why UHC? |
| Atun R et al/2015 | Health system reform and UHC: Mixed method analysis | Latin America | Why UHC? |
| Ozano K et al/2020 | A call to action for UHC: viewpoints | Not specific | Why UHC? |
| Feng XL et al/2022 | Progress towards UHC: Quantitative study | China | Why UHC? |
| Fisher M et al/2022 | UHC for non-communicable diseases and health equity: Qualittaive study | Australia | Why UHC? |
| Atun R et al/2013 | UHC: method Not clearely specified | Turkey | Why UHC? |
| Aungkulanon S et al/2016 | Post UHC trend and inequalities of mortality: Quantitaive study | Thailand | Why UHC? |
| Bayked AM et al/2023 | The impact of community-based health insurance on UHC: systemati review & meta-analysis | Ethiopia | Why UHC? |
| Ranabhat CL et al/2020 | Structural factors responsible for UHC: Quantitative study | Low- and middle-income countries | Why UHC? |
| Takura T & Miura H/2022 | Socioeconomic determinants of UHC: Quantitative study | Asia | Why UHC? |
| Taniguchi H et al/2021 | Equity and determinants in UHC indicators: Quantitative study | Iraq | Why UHC? |
| Wenham C et al/2019 | Global health security and UHC: concept analysis | Not specific | Why UHC? |
| Hirose N et al/2022 | UHC before and after earthquake: Quantitative study | Haiti | Why UHC? |
| Assefa Y et al/2020 | Global health security and UHC: Quantitative study | worldwide | Why UHC? |
| Lee Y et al/2022 | An association between international health regulation and UHC: Quantitative study | worldwide | Why UHC? |
| Lal A et al/2022 | Pandemic preparedness and response: the role of UHC: methos not specified | Not specific | Why UHC? |
| Tadiosi F et al/2016 | BRICS countries and the global movement | Not specific | why UHC? |
| Venkateswaran S et al/2022 | Political motivation as a key driver for UHC: systematic review | India | Why UHC? |
| Mao W et al/2020 | Advancing UHC: mixed-method study | China and Vietnam | Why UHC? |
| Eusebio C et al/2023 | How to achieve UHC? Commentary | Uganda | How and Why UHC? |
| Greer SL et al/2015 | UHC: a political struggle and governance challenge: Commentary | Not specific | Why UHC? |
| Srivastava S et al/2023 | The genesis of the PM-JAY health insurance scheme: Qualitative study | India | Why UHC? |
| United Nations/2019 | Political declaration of the high-level meeting on UHC: Meeting report | UNs member state | Why UHC? |
| Chemouni B/2018 | The political path to UHC | Rwanda | Why UHC? |
| Meneses Navarro S et al/2022 | Health system segmentation: short communication | Mexico | Why UHC? |
| Landrum KR et al/2022 | Pediatric surgical financing and UHC: Qualitative analysis | Guatemala | Why UHC? |
| Stern A/2008 | An American partnership for UHC: perspective | USA | How UHC works? |
| Kurniati A et al/2015 | Partnership for health workforce development and UHC: Document analysis | Indonesia | How UHC works? |
| Wood A/2023 | Partnership, community health insurance, and UHC: Qualitative study | Senegal | How UHC works? |
| Machida M & Miyashita Y/2021 | Partnership towards UHC: policy forum | Japan and Thailand | How UHC works? |
| Tambo E et al/2017 | China-Africa health development initiatives: method not clearly specified | China and Africa | How UHC works? |
| Smithers D & Waitzkin/2022 | Political hegemony of UHC: mixed method study | Low- and middle-income countries | Why UHC? |
