# Supplementary material for: Universal health coverage—Exploring the what, how, and why using realist review
Source: PLOS Glob Public Health. 2025 Mar 18;5(3):e0003330. doi: 10.1371/journal.pgph.0003330 (PMC11918392; doi:10.1371/journal.pgph.0003330)
Supplement: S3 File — (DOCX) [file pgph.0003330.s003.docx]

| **S3 File: Australia’s Public Health Insurance system as a case study for UHC**  **Medicare** is Australia’s universal health insurance scheme. It covers all Australian and New Zealand citizens, Australian permanent residents, those who have applied for permanent residency (conditions apply), temporary residents under a ministerial order, and citizens or permanent residents of Norfolk Island, Cocos Islands, Christmas Island, Lord Howe Island, as well as individuals from countries with a reciprocal healthcare agreement. **Services covered by Medicare** are hospital services, private hospital (Medicare covers some of the doctor’s fee but not accommodation and theatre fees), medical services (consultations with health practitioners, like general practitioners, specialists and other health practitioners; mental health services, including assessment, treatment plans and medicines; and some dental procedures, under the Child Dental Benefits Schedule), imaging and scans (magnetic resonance imaging, nuclear medicine scans like position emission tomography, ultrasounds, computed tomography scans, X-rays), pathology tests (blood, urine or tissue), eye tests (once every 3 years for under 65 years and once a year for 65 years or older), medicines via Pharmaceutical Benefits Scheme, and the National Immunisation Programme covers the cost of vaccines. **Services not covered by Medicare** are ambulance services, most dental services, glasses and contact lenses, hearing devices, elective and cosmetic surgery, services not on Medicare Benefit Scheme lists, services provided through private health system, and Medicare does not work overseas. **Supports from other programmes specific to selected services:** Breast Screen Australia program, Closing the Gap Pharmaceutical Benefit Scheme Co-payment Program, Continence Aids Payment Scheme, Department of Veternars’ Affairs Optical services, Hearing services programme, Life Saving Drugs Programme, Medical treatment overseas programme, National Bowel Cancer Screening Programme, National Cervical Screening Program, National Diabetes Services Scheme, Pharmaceutical Benefits Scheme, Stoma Applicants Scheme, Visiting Optometrist Scheme, State and territory-based schemes for glasses and contact lenses. **Costs covered by Medicare**: Medicare covers all public hospital costs. It considers100% reimbursement or pay the difference if clients paid higher than fee list under Medicare Benefit Scheme. Medicare covers 75% of costs if clients attend private hospital. Medicare pays 85% of the schedule fee for a specialist and 100% for general practitioners. **Medicare’s sources of finance**: Income tax, including the Medicare levy and the Medicare levy surcharge. **Medicare Safety Net**: The Medicare Safety Net can help keep them under control if you have a lot of out of hospital costs. If the costs for the year reach a threshold amount, Medicare Safety Net will pay more of the costs. **Concession cards and PBS Safety Net**: Concession cards provide further discounts on Pharmaceutical Benefits Scheme medicines. Pharmaceutical Benefits Scheme Safety Net covers excess costs if clients need many medicines or pass the yearly threshold. **Social health protection**: assist people to meet their basic needs such as food, transport service Via Centrelink. **Private health insurance**: Cover health care costs that Medicare and Pharmaceutical Benefits Scheme do not. |
| --- |
|  |
|  |
|  |
|  |
|  |
|  |
|  |
|  |
|  |
|  |
|  |
