# Supplementary material for: Universal health coverage—Exploring the what, how, and why using realist review
Source: PLOS Glob Public Health. 2025 Mar 18;5(3):e0003330. doi: 10.1371/journal.pgph.0003330 (PMC11918392; doi:10.1371/journal.pgph.0003330)
Supplement: Attachment — Submitted filename: Reviewer XXX PLOS GLOBAL PUBLIC HEALTH.docx [file pgph.0003330.s005.docx]

Reviewer XXX

PLOS GLOBAL PUBLIC HEALTH

PLOS Global Public Health

**Universal Health Coverage – Exploring the What, How, and Why Using Realistic**

**Review**

**Good areas**

The paper is interesting reading about Ethiopian community-based health insurance and the issues of reluctance to renew their membership to the community-based scheme. I have learnt and gain some useful insights on the study.

The discussion section is well done and its far better than the presentation of findings

**Areas that need some clarity and revision**

It is not clear if the two words or areas mean the same in the paper.

1. The main purpose and the problem of investigation should be stated clearly in the abstract and in the introduction.
2. In the title it is **realistic review**, in the keywords, it is **realist review**. The authors may clarify the two terms or words
3. I am not sure if the authors used systematic review or not, also if PRISMA was part of their search for secondary data. As stated on page 5: “We searched PubMed, SCOPUS, EMBASE, and Web of Science. Additionally, we accessed Google Scholar, Google, WHO website, UNs, UNIECF, and the WB website to retrieve unpublished work, grey literature, and guidelines or appropriate reports. We also checked the reference lists of eligible articles for further inclusion”.
4. These words should not be Bold from pages 12-13 example Value-Based Health Care…
5. The study findings are loosely presented, and I must say the findings are not well-done.

I suggest the authors present the findings along some major themes rather that the multiple sub-themes, which are too clumsy. The style of presentation of the findings should be revised and presented with some major themes.

Reviews XXX Recommendation

**Accept submit to revision**

September 2024
