# Supplementary material for: Universal health coverage—Exploring the what, how, and why using realist review
Source: PLOS Glob Public Health. 2025 Mar 18;5(3):e0003330. doi: 10.1371/journal.pgph.0003330 (PMC11918392; doi:10.1371/journal.pgph.0003330)
Supplement: Attachment — Submitted filename: Response to reviewers.docx [file pgph.0003330.s007.docx]

Submission ID: PGPH-D-24-01157
Title: **Universal Health Coverage – Exploring the What, How, and Why Using Realist Review
Dear Editor and Reviewer,**

We appreciate your support in handling and reviewing our manuscript. In response to the reviewer’s feedback, we carefully revised the manuscript and prepared a comprehensive rebuttal letter outlining a point-by-point response letter to address the reviewers’ comments, suggestions and questions. Our responses are presented in italic style and underlined.

Reviewer #1: The article is too descriptive, and contents in the main findings can be shorten, and write more concisely than the submitted manuscript. The manuscript looks like a chapter of Master degree or PhD thesis. It is unclear about the screening process to exclude a number of literature (n=13,993) using the title and abstract of the records. In addition, the discussion section should be written concisely and clearer than the draft manuscript.

*Authors’ response: We appreciate your review of our manuscript. As you noted, our review addresses what, how, and why of UHC. These questions require qualitative synthesis and presentation through describing and explaining concepts and interrelated terms. This is why the word count may seem high compared to most quantitative studies and systematic reviews. If we do not explain how the identified strategies relate to UHC and its relevance, we risk presenting a manuscript that falls short by not adequately addressing key concepts and clarifications. Yes, we have revised the manuscript to address the comments raised during the review process. Regarding article screening processes, we thoroughly followed PRISMA flow chart as presented in Figure 1. For instance, 13,993 were excluded because the title and abstract did not address the what, how, and why of universal health coverage, including titles that did not mention universal health coverage.*

**Authors’ response**:

Reviewer #2: Revise based on reviewer comments
Good areas
The paper is interesting reading about Ethiopian community-based health insurance and the issues of reluctance to renew their membership to the community-based scheme. I have learnt and gain some useful insights on the study.
The discussion section is well done and its far better than the presentation of findings

*Authors’ response: Thank you for the appreciation.*
Areas that need some clarity and revision
1. The main purpose and the problem of investigation should be stated clearly in the abstract and in the introduction.

*Authors’ response: This realistic review was conducted to fill the knowledge gap by synthesising evidence on the meaning, components, significance, and strategies of UHC*.
2. In the title it is realistic review, in the keywords, it is realist review. The authors may clarify the two terms or words

*Authors’ response: Thank you for your comment. We have corrected it to indicate that we mean 'Realist Review’.*
3. I am not sure if the authors used systematic review or not, also if PRISMA was part of their search for secondary data. As stated on page 5: “We searched PubMed, SCOPUS, EMBASE, and Web of Science. Additionally, we accessed Google Scholar, Google, WHO website, UNs, UNIECF, and the WB website to retrieve unpublished work, grey literature, and guidelines or appropriate reports. We also checked the reference lists of eligible articles for further inclusion”.

*Authors’ response: As you know, different types of reviews exist for different purposes. Our review is a realist review, but we searched for, gathered, and reviewed articles systematically. We utilized the PRISMA flow chart to screen the articles. In the revised manuscript, we updated this section to read: After collecting the articles, we followed the PRISMA flow chart to select eligible articles and excluded those that irrelevant to UHC.*
4. These words should not be Bold from pages 12-13 example Value-Based Health Care…

*Author response: We have corrected them in the revised submission.*
5. The study findings are loosely presented, and I must say the findings are not well-done.
I suggest the authors present the findings along some major themes rather that the multiple sub-themes, which are too clumsy. The style of presentation of the findings should be revised and presented with some major themes.

*Authors’ response: As you know, a realist review requires an explanation to uncover the strategies in achieving the desired outcomes. We have identified eight major themes, each of which stands independently and cannot be categorized into smaller themes. Each major theme has its own subthemes. The mechanisms of action for each subtheme should be explained, as this strengthens the realist review; understanding the 'how' is one of the key questions addressed in the review.*
Reviewer #3

The paper does a good job of bringing together and summarizing the meaning and scope of Universal health coverage and the strategies required to achieve it. UHC is easy to say but hard to implement comprehensively. This paper reviews the meaning, components, significance, and strategies of UHC, which will help researchers and policymakers better understand it. Some revision should be taken in this paper.

*Authors’ response: Thank you so much for the compliment.*
Introduction
- Some sentences are repeated: (This will provide detailed understanding of UHC, the reasons and significance and relevance of UHC, as well as the mechanisms towards UHC.)/(This article aims to synthesis the evidence on the meaning of UHC, its components, its significance, and implementation strategies to achieve it..).

*Authors’ response: We have revised this in the revised submission. To read: We argue a realist review is timely to inform UHC policy and implementation, highlighting its significance and mechanisms.*
- There is incoherence between the claims made in the introductory of the paper and the main take that I derived. For example, in Paragraph 5 the authors claim that ‘We argue that it is timely to systematically and realistically review and synthesize the available evidence to guide policy and implementation of the UHC agenda.’ but the implementation strategies and action plans are not explicitly spelt out in the paper. This is very important, because health systems in different contexts are at different maturity levels pursuing different objectives related to their priorities.

*Authors’ response: In the introduction, we stated, 'We argue a realist review is timely to inform UHC policy and implementation, highlighting its significance and mechanisms.' We mean that conducting a realist review on the what, how, and why will equip policymakers and practitioners with clear information to design and implement strategies effectively. Our review results outline strategies in the 'how' section of the realist review.*
-The Introduction section could be made stronger by acknowledging health financing. For example, in Paragraph 5, other than the mentioned research publications like “measurement and attainment of UHC for older people and people with stroke, there are a number of research publications on health financing of UHC.

*Authors’ response: We agree with your suggestions and have included health financing of UHC in the revised version.*
Method:
- As there are few published examples of realist synthesis, please verify your reason for conducting a realist synthesis and discuss its focus.

*Authors’ response: We agree that there were different reviews with distinct purposes. For instance, evolution and future challenges of UHC, and the processes and evidence of UHC. These articles do not replace the current realist review. While some identified challenges and facilitators towards UHC, they did not address the broader mechanisms or the necessity for launching UHC. We have provided more information in the introduction section of the revised submission.*
-In “Search for evidence” section, the authors claim that they have included materials without restriction on language. But, in “Limitation of this review section”, they mention that only articles published in English have been included.

*Authors’ response: Thank you so much for your detail review. We have revised it as we only included articles published in English in the revised submission as to read: The articles eligible for inclusion in the study were published in English.*
Discussion
It would be helpful to cast light on the UHC in middle- income countries. The countries selected to be discussed are either well- developed or less developed countries.

*Authors’ response: We have included in the revised submission that some middle-income countries, such as Thailand and Chile, have achieved better UHC by implementing new initiatives.*
Minor comments
-Consider introducing the policy implications section in the discussion

*Authors’ response: We added implication sections in the revised submission as follow: Regarding implications, healthcare institutions and health education centers play a role in translating the concepts and purposes of UHC. Adopting effective mechanisms that foster UHC and enhance local population engagement in planning and implementation is essential, ensuring that services are culturally acceptable and appropriate. It is also crucial to monitoring and evaluation activities on the implementation status of UHC initiatives, identify weaknesses and challenges, and align broader concepts, programs, and policies with social and economic strategies to tackle the social determinants of health, ensure health security, achieve health equity*.
- Please write the full word for these abbreviations: RMNCH and CHE. (Reproductive, Maternal, Newborn, and Child Health)/ (Catastrophic Health Expenditures).

*Authors’ response: Thank you for this comment. We have considered in the revised submission.*
